# Dynamics of Bacterial and Viral Communities in Paddy Soil with Irrigation and Urea Application

**DOI:** 10.3390/v11040347

**Published:** 2019-04-16

**Authors:** Yuting Li, Hao Sun, Weichao Yang, Guanxiong Chen, Hui Xu

**Affiliations:** 1 Institute of Applied Ecology, Chinese Academy of Sciences, Shenyang 110016, China; liyuting211@mails.ucas.ac.cn (Y.L.); haos@spaces.ac.cn (H.S.); yangweichao@iae.ac.cn (W.Y.); gxchen39@iae.ac.cn (G.C.); 2 University of Chinese Academy of Sciences, Beijing 100049, China; 3 National Field Observation and Research Station of Shenyang Agro-Ecosystems, Shenyang 110016, China; 4 Key Laboratory of Pollution Ecology and Environmental Engineering, Institute of Applied Ecology, Chinese Academy of Sciences, Shenyang 110016, China

**Keywords:** soil virus, soil bacteria, viromes, paddy soil, viral regulation, soil nutrient cycling

## Abstract

Viruses are ubiquitous in natural systems. By influencing bacterial abundance (BA) and community structure through lysis-lysogenic conversion, viruses are involved in various ecological processes. In agricultural management, nitrogen addition and irrigation should be considered as important factors that can modify soil viral dynamics but have been ignored. In our study, short-term dynamics of autochthonous soil viral and bacterial abundance and diversity after irrigation and urea application were examined in a long-term experimental paddy field. Urea addition delayed the emergence of peak viral abundance for three days, suggesting that viruses are sensitive to N addition. Under short-term eutrophic conditions through urea application, viruses undertake a lysogenic-biased strategy. Moreover, nitrogen-fixing bacteria were most likely specifically lysed in urea-treated soil, which suggests that soil viruses block N accumulation by killing nitrogen-fixing bacteria. To the best of our knowledge, this study is the first to investigate dynamic changes in autochthonous viruses in paddy fields.

## 1. Introduction

There has been a surge of interest in the past few decades in the roles that viruses play in the environment [1]. Viruses are ubiquitous and numerous (ca. 10^31^) and parasitize various organisms. Viruses constitute a repository of genetic diversity and greatly influence the ecosystem structure and function [2,3]. According to previous studies on freshwater and marine environments, viruses can mediate the essential processes of nutrient biogeochemical cycling [4,5,6], manage microbial community composition through lysis and lysogeny oscillation [5,7,8,9], and are involved in host evolution via the horizontal gene exchange [1,3].

As a sharp contrast to the growing number of studies on viruses in aquatic environments, there has been a relatively slow progress on research of soil viruses. One of the main obstacles is the high heterogeneity of soils, because it limits our ability to isolate, resuspend soil viruses, and extract their DNA [10,11]. Most soil viral ecology studies focus on natural systems and not artificial soils like agricultural soils, e.g., hot deserts [12,13], active layer above permafrost [14,15], and polar regions [16,17]. Agricultural ecosystems not only provide a large amount of food for humans, but also affect the environment through processes such as nitrogen leaching, ammonia volatilization, and CH_4_ and N_2_O emissions [18]. In addition, unlike aquatic and other terrestrial ecosystems, agricultural ecosystems are also severely disturbed by agricultural activities such as fertilization, irrigation, and tillage. Therefore, the role of viruses in biogeochemical cycling of agricultural ecosystems may be completely different from those in aquatic ecosystems [19,20,21].

In agricultural soil-based studies, most observations on autochthonous viruses have been performed in dry soils [19,20,21], while paddy soils have long been neglected. However, in the paddy soil, wet–dry cycling changes soil pH and nutrient availability [22,23], and also promotes the level of connectivity across aggregates, and thus increases the chance of microbial encounters [10]. Recent studies have demonstrated that both soil water content (SWC) and pH influence virus–bacterium interactions, thus affecting the dynamics of viral and bacterial communities in soils [10,11,24]. Moreover, it has been reported that viruses are also sensitive to changes in soil organic material (SOM) [20], thus specifically modifying their host population [5]. Furthermore, previous reports about soil viruses have discussed the impact of different carbon substrates on viral communities [19,25]. However, the effect of nitrogen addition has been neglected. The addition of the chemical nitrogen fertilizer is unavoidable in agriculture; therefore, it is meaningful to understand how viruses respond to nitrogen fertilization and further regulate bacterial populations.

Owing to the limited knowledge regarding autochthonous viruses in agricultural soils and the likely important roles they play in microbial biogeochemistry, it is important to better characterize autochthonous viruses in paddy soils. We investigated the dynamics of viral and bacterial communities, under flooding and fertilization conditions, and the effect of multiple environmental factors on soil viruses at the beginning of the spring ploughing season from a long-term (since 1991) urea fertilized paddy field in northeastern China.

## 2. Materials and Methods

### 2.1. Field Description and Experimental Design

This study was carried out at the National Field Observation and Research Station of Shenyang Agro-ecosystems (41°31’N, 123°24’E). The site has a temperate semi-humid continental monsoon climate, with a mean annual average temperature of 7.7 °C and average precipitation of 675 mm year^−1^. The soil is classified as a Cambisol (FAO taxonomy) [26] and the cropping system used is a single cropping of rice.

The long-term fertilization experiment commenced in 1991 and two treatments (five plots each, with an area 4 m × 6 m) were established: a) Phosphate and potassium fertilizer (PK), b) phosphate, potassium, and nitrogen fertilizer (PKN). The amount of fertilizer application for both treatments simulated the field management practices of farmers. In the experimental period, two treatments were applied in the same amount of calcium superphosphate (90 kgP ha^−1^) and potassium chloride (90 kgK ha^−1^); in addition, the PKN group received urea at a rate of 60 kgNha^−1^. When starting irrigation, the fields maintained a flooded condition during the sampling period. To maintain the water level at ~5 cm high during sampling, the irrigation was implemented daily at 7:00 a.m.

### 2.2. Sample Collection and Soil Analysis

**Figure 1:**
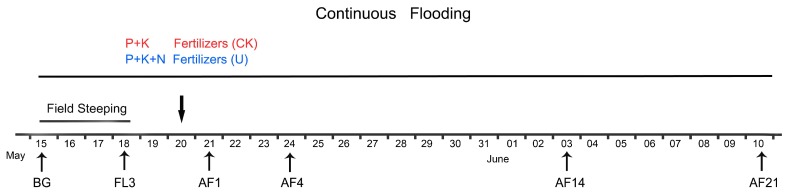
Detailed description of water management and fertilizer application in the experimental fields, and each sampling date. The downward facing arrow indicates that the application of fertilizers was performed on May 20th. Soil samples representing spring fallow period (FP) collection and starting irrigation were performed on the same day. Irrigation began immediately after FP samples collection. FL3, third day of flooding; AF1, 4, 14, 21 refer to the first, fourth, 14th, and 21st days after fertilization, respectively.

Soils were collected in 2018 on the following dates: May 15 for obtaining fallow period values (FP), May 18 for completing the field steeping period, namely the third day of flooding (FL3), May 21, which is the first day after fertilization (AF1), May 24 (AF4), June 3 (AF14), June 10 (AF21) (Figure 1). At 9:00 a.m. on each date, 10 cores (diameter, ~3 cm; depth, 0–10 cm) were taken from each replicate plot of every treatment and homogenized separately by mixing, stored in sterile bags, and transported to the laboratory on ice. It should be noted that each group had its independent core device with a replaceable cutting bit. Those cutting bits were cleaned and sterilized before sampling. Every cutting bit was replaced after sampling a soil core. Soil viral and bacterial extractions, soil total DNA extraction, and gaseous emission measurements were performed immediately. Soil physicochemical properties (Table 1) were determined following the methods of Lu [27]. Data on precipitation and temperatures (depth, 5 cm) were acquired from the meteorological station of the National Field Observation and Research Station of Shenyang Agro-Ecosystems (http://sya.cern.ac.cn/meta/metaData).

### 2.3. Samples Processing

The soil viral extraction method was previously described by Williamson et al. [28]. Briefly, raw soil samples (5 g) were suspended in 15 ml 1% potassium citrate extraction buffer (containing per liter 10 g K_3_C_6_H_5_O_7_·H_2_O, 1.44 g Na_2_HPO_4_·7H_2_O, 0.24 g KH_2_PO_4_, pH 7), vortexed vigorously, and then placed in a shaker at 180 rpm, 4 °C, for 1 h. After centrifuging at 2000× *g* for 45 min at 4 °C, the supernatants were filtered through 0.22-μm filters (Merck Millipore Ltd, Tullagreen, Carrigtwohill, Ireland). Finally, the extracts were snap-frozen in liquid nitrogen and stored at −80 °C.

Bacteria were extracted according to Williamson et al. [29]. A mixture of 5 g soil and 35 mL 1% potassium citrate extraction buffer was vortexed and then shaken on ice for 1 h. Prior to centrifugation at 10,000× *g* for 20 min at 4 °C, the slurry was layered over an 8-mL layer of Nycodenz (1.3 g mL^−1^ density, filtered through a sterile 0.22-μm filter before use). After centrifugation, the supernatant was snap-frozen in liquid nitrogen and stored at −80 °C.

### 2.4. Epifluorescence Microscopy

The counts of virus-like particles (VLPs) and bacterial cells were performed as previously described [20,30]. Briefly, DNase-treated (RNase-free recombinant DNase I, Takara Bio, Kusatsu, Japan) and 100-fold diluted viral aliquots and bacteria aliquots were respectively passed through a 0.02-µm pore Anodisc filters (diameter, 25 mm; Whatman, Dassel, Germany) and 0.2-µm pore Nuclepore Track-Etch membranes (Whatman, Maidstone, UK). Filters were immediately stained with 25× SYBR Safe (Invitrogen, Carlsbad, CA, USA) before being incubated in the dark for 20 min. Dried filters were put onto glass slides with 30 μL anti-fade mounting medium, a mixture of 0.1% (vol/vol) p-phenylenediamine, 50% glycerin, and 50% PBS (0.13 mol/L NaCl, 7.0 mmol/L Na_2_HPO_4_, 3.0 mmol/L NaH_2_PO_4_), then VLPs or bacterial cells were enumerated using epifluorescence microscopy under blue light excitation. What needs to be stated is that specific bacterial probes have not been applied in bacterial counting. Thus, we are not 100% sure only bacteria cells were specifically counted on the track-etch membranes, which may slightly influence the accuracy of bacterial abundance (BA) and virus-to-bacterium ratio (VBR).

### 2.5. Total DNA Extraction, Sequencing of 16s rDNA, and Bioinformatics Analysis

DNA was extracted from 0.4 g (wet weight) soil using the DNeasy PowerSoil DNA extraction kit (Qiagen, Hilden, Germany, product 12888) with three replicates, according to the manufacturer’s instructions. The isolated DNA was eluted with 80 μL of solution C6 (Qiagen, Hilden, Germany, product 12888). Before storing at −20 °C, the total DNA was checked on 1.0% agarose gel and the DNA quantity and quality of the extracts were estimated by the spectrophotometric analysis (Bio-Photometer, Eppendorf, Hamburg, Germany). All samples of the DNA were amplified in triplicate with no-template controls and detected by agarose electrophoresis. The v3 and v4 regions were amplified using primers containing the sequences 5′-CCTACGGRRBGCASCAGKVRVGAAT-3′ and 5′-GGACTACNVGGGTWTCTAATCC-3′, which were designed by GENEWIZ, Inc. (Suzhou, China). Three replicates of purified PCR amplicons were used to create the sequencing libraries. All libraries were generated using the TruSeq® DNA PCR-Free Sample Preparation Kit (Illumina, San Diego, CA, USA) following the manufacturer’s recommendations, and barcodes were added. Lastly, the libraries were sequenced on an Illumina MiSeq 300PE platform at GENEWIZ, Inc. (Suzhou, China).

The sequencing reads were merged using the FLASH tool, and the quality filtering on raw tags was performed under specific filtering conditions according to QIIME (QIIME™, V2.0) [31]. Chimeric sequences were wiped off using USEARCH. High-quality sequences from all samples were clustered into operational taxonomic units (OTUs) at 97% sequence similarity using the default QIIME pipeline UCLAST. The representative sequence in each OTU was assigned to a taxonomic group using the SILVA classifier with an 80% confidence threshold, thus establishing the OTU table [32].

### 2.6. Randomly Amplified Polymorphic DNA (RAPD)-Polymerase Chain Reaction

The RAPD-PCR technique was performed to get at viral diversity [33]. The viral concentration method was adapted from that described by Srinivasiah et al. [33]. Briefly, soil viral extracts were spun at >200,000× *g* at 4 °C for 2 h (41,000 rpm/288,000× *g* in an SW 41 Ti rotor; Beckman Coulter Inc., Fullerton, CA, USA). The precipitate was resuspended in 20 μL 1% potassium citrate extraction buffer and then the concentrate was treated with 5 units of RNase-free recombinant DNase I (Takara) to remove any free DNA following the manufacturer’s protocol.

The RAPD-PCR assay in this study involved two successive rounds. In the first round, the reaction mixture (25 µL) included 12.5 µL 2X Taq Plus PCR Master Mix (Tiangen, Beijing, China) containing 500 μM dNTP each, 20 mM Tris-HCl, 100 mM KCl, 3 mM MgCl_2_, and 4 µL template (about 10^6^ VLPs), 6.5 µl ddH_2_O, and 2 µL of 10 µM AAWZS-1 (5’-CACCACCTGC-3’) [33] as both forward and reverse primer. RAPD-PCR amplifications were conducted in a Biometra TGradient thermocycler (Biometra, Göttingen, Germany) as follows: after 6 min initial denaturation at 95 °C, 40 cycles were carried out at 95 °C for 1 min, 41 °C for 3 min, 72 °C for 2 min, and then the final extension was 20 min at 72 °C. During the second round, the 25 µL reaction volume contained 12.5 µL 2× Taq Plus PCR Master Mix, 1 µL amplified fragments as template, 2 µL primer AAWZS-1, and 9.5 µL ddH_2_O. The RAPD-PCR conditions were 94 °C for 5 min, 30 cycles consisting of 94 °C for 1 min, 43 °C for 1.5 min, and 72 °C for 2 min for extension, followed by 72 °C for 15 min. Amplified products were analyzed using an automated QIAxcel Advanced electrophoresis system (QIAGEN, Hilden, Germany).

### 2.7. Gaseous Emissions Measurements

Gas emissions (CH_4_, CO_2_, and N_2_O) were analyzed as follows. About 100 g soil was put in a glass bottle (300 mL) at 20 °C, and soils that were collected under continuous flooding were maintained with a 1 cm water height. Each treatment had five replicates. After incubating for ~4 h, gas samples (35 mL) in the headspace were taken and analyzed simultaneously with a gas chromatograph (Agilent 7890A, Wilmington, DE, USA), equipped with an electron capture detector and a flame ionization detector. Gaseous emissions were calculated with the following equation:

F = ∆m/(W × ∆t) = ρ × V × ∆c/(W × ∆t),

where F is the CH_4_, CO_2_, or N_2_O fluxes; ∆m and ∆c are the increase and decrease of gaseous mass, and concentration ratio in bottled gas mixtures, respectively; V and W are the volume of effective space and soil dry weight, respectively; ∆t is the cultivation time; ρ is gas density under standard conditions.

### 2.8. Statistical Analysis

Independent sample *t*-test and spearman’s rank correlation analysis were performed using the SPSS 21.0 software (SPSS Inc., Chicago, IL, USA). Bacterial Shannon index, and unweighted UniFrac values were calculated with QIIME 2 (QIIME™, V2.0) and visualized with the “phyloseq” package (version 3.7) in R (version 3.4.3) [34]. Principal component analysis (PCA) of the viral community was performed using the “vegan” package in R according to the RAPD electrophoresis pattern. Principal coordinate of analysis (PCoA) was performed with the “phyloseq” package in R according to the bacterial UniFrac distance matrix. Top phylum and genus levels were counted and analyzed using the GraphPad Prism 6 (GraphPad Software Inc., San Diego, CA, USA). Redundancy analysis (RDA) was performed using the vegan package in R.

## 3. Results

### 3.1. Viral and Bacterial Abundance

**Figure 2:**
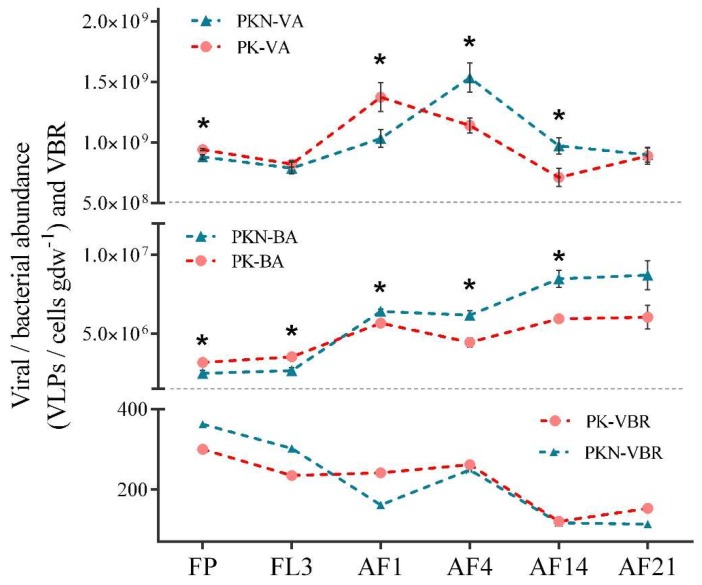
Dynamics in viral abundance (VA), bacterial abundance (BA) and virus-to-bacterium ratio (VBR) in the phosphate and potassium fertilizer (PK) and phosphate, potassium, and nitrogen fertilizer (PKN) groups. The asterisks indicate significant differences are obtained from matched samples (*T*-test; *, *p* < 0.05). FP, representing spring fallow period measurements; FL3, third day of flooding; AF1, 4, 14, 21 refer to the first, fourth, 14th, and 21st days after fertilization, respectively.

Viral abundance (VA) and bacterial abundance from the samples collected from the day before irrigation (FP) to the 21st day after fertilization (AF21) were observed by using epifluorescence microscopy. VA in the PK group ranged from 7.12 × 10^8^ to 1.37 × 10^9^ VLPs gdw^−1^, and in the PKN group ranged from 7.88 × 10^8^ to 1.54 × 10^9^ VLPs gdw^−1^ (Figure 2, Supplementary Materials Table S1). These results were similar to those from a previous study that summarized the existing studies on dry farmland, and found a VA range of ~10^7^ to 10^9^ gdw^−1^ [11]. In our study, BA ranged from 3.17 × 10^6^ to 6.04 × 10^6^ cells gdw^−1^ in the PK group and 2.49 × 10^6^ to 8.71 × 10^6^ cells gdw^−1^ in the PKN group. However, according to the above-mentioned study [11], BA values were mostly around 10^9^, which were much higher than those in the present study. It is presumed that the higher BA levels in soils in those previous studies were the result of organic fertilizer application. The FP samples reflected the effect of long-term urea application on paddy soils. After about 30 years of fertilization, VA and BA in the PKN group were both significantly lower than those in the PK group (VA, *p* = 0.019; BA, *p* = 0.014) (Figure 2, Supplementary Materials Table S1). These results indicate that the long-term urea addition led to a significant difference in VA and BA in the paddy soil during the spring fallow period.

For the FL3 samples, VA in the PK group was significantly decreased by flooding (*p* = 0.003), while the abundance in the PKN group was not significantly different (*p* = 0.105). The difference in VA between the two groups disappeared by FL3 (*p* = 0.542). The large change in SWC led to an increase in BA in both groups, and the abundance in the PK group was still significantly higher than that in the PKN group in the FL3 samples (*p* = 0.005) (Figure 2, Supplementary Materials Table S1). From FL3 to AF1, VA under the two treatments increased likely due to fertilization. In the PK group, VA sharply increased to 1.37 × 10^9^ viruses gdw^−1^ after the first day of fertilization, then fell to the minimum value of 7.12 × 10^8^ VLPs gdw^−1^ at AF14. In the PKN group, VA increased from an initial value of 1.03 × 10^9^ to 1.54 × 10^9^ VLPs gdw^−1^ at AF4, while it decreased to 8.98 × 10^8^ VLPs gdw^−1^ at AF21 (Figure 2, Supplementary Materials Table S1). BAs in the two groups increased after the first day of fertilization. Besides, BA in the PKN group was significantly higher than that in the PK groups at AF1 (*p* = 0.015), AF4 (*p* = 0.003), and AF14 (*p* = 0.003). In the PK group, BA reached 5.67 × 10^6^ cells gdw^−1^ at AF1, then significantly decreased at AF4 (*p* = 0.015), and finally rose to 6.04 × 10^6^ cells gdw^−1^ at AF21. In the PKN group, BA increased from an initial value of 6.42 × 10^6^ to 8.71 × 10^6^ cells gdw^−1^ at AF21.

The highest values of VBR were observed in the FP samples of both groups at 300.23 and 362.53 in PK and PKN groups, respectively (Figure 2, Supplementary Materials Table S1). Subsequently, downward trends were observed in both groups for the rest of the experimental period. The lowest VBR values were observed in the PK group at AF14 (120.49) and the PKN group at AF21 (107.20) (Supplementary Materials Table S2).

### 3.2. Viral and Bacterial Community Analyses

**Figure 3:**
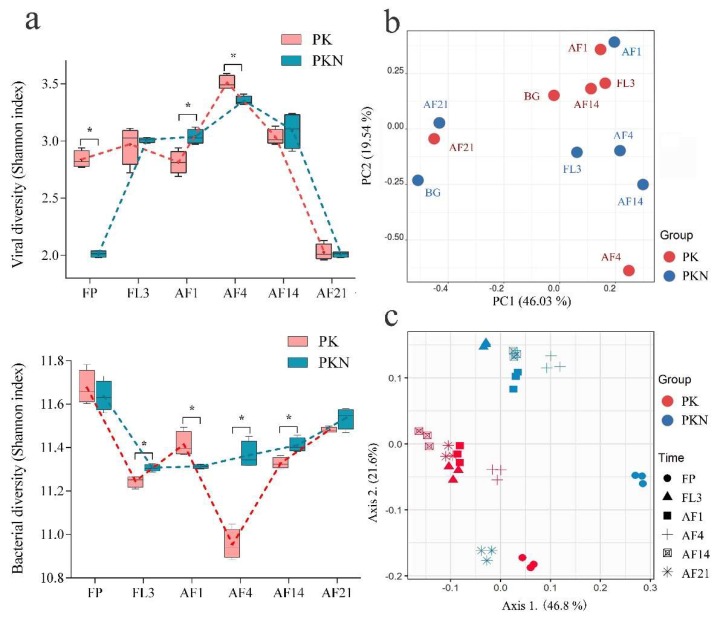
Changes in viral and bacterial communities over time in the PK and PKN groups. (**a**) Viral (top) and bacterial (bottom) diversities shown as Shannon index in all samples. The asterisks show the significance determined by independent sample *t*-test (*, *p* < 0.05); (**b**) PCA analysis shows the shifts of the viral community in paddy soil; (**c**) scatterplots from PCoA, based on unweighted UniFrac distances of the OTUs in all samples at each of six time points. FP, representing spring fallow period; FL3, third day of flooding; AF1, 4, 14, 21 refer to the first, fourth, 14th, and 21st days after fertilization, respectively.

Variation in viral communities in paddy soil was detected with RAPD-PCR and high-resolution capillary electrophoresis techniques. Results with primer AASW1 and its amplifying system were more reproducible than those from a previous microcosmic study of dry soil (Supplementary Materials Figure S1) [25]. Long-term urea addition significantly decreased viral diversity in FP of the PKN group (*p* = 0.000), of which the Shannon index was 2.01, lower than the 2.84 in the FP samples of the PK group (Figure 3a, Supplementary Materials Table S2). A sharp increase in viral diversity in the PKN group was observed at FL3 (average of 2.01 in FP of the PKN group to 3.01 in FL3 of the PKN group, *p* = 0.000), while a similar trend was not found in the PK group (average from 2.84 in FP of the PK group to 2.97 in FL3 of the PKN group, *p* = 0.207). Consequently, no difference in viral diversity between the two groups was observed at FL3 (*p* = 0.683) (Figure 3a).

Within 21 days after fertilization, dynamic trends in the viral diversity of the two groups were almost the same (Figure 3a). At AF1, the diversity did not significantly change in either group (PK, *p* = 0.165; PKN, *p* = 0.439), which demonstrated that fertilization did not rapidly trigger a large variation in viral diversity under the two treatments. Furthermore, at AF1, viral diversity in the PKN group was significantly higher than that in the PK group (*p* = 0.009). Viral diversity peaked in the soils under both treatments at AF4 (3.51 in the PK group and 3.35 in the PKN group), and in contrast to that at AF1, the diversity in the PKN group was significantly lower than that in the PK group at AF4 (*p* = 0.005). In the last two observational days, viral diversity was not different between the two groups (AF14, *p* = 0.487; AF21, *p* = 0.647) (Supplementary Materials Table S2). Meanwhile, the viral diversity of both treatments decreased to its lowest point at AF21 (Figure 3a).

The PCA results, based on electrophoretogram analysis, indicated that the soil moisture and urea addition caused divergence in viral communities (Figure 3b). Viral communities in the FP samples of both groups were separated along the first principal component axis (PC1) explaining 46.03% of the variation in the data, suggesting that the soil viral community structure was strongly affected by the long-term urea application. Along the second principal component axis (PC2), explaining 19.54% of the variation, the viral communities of the PK group at AF4 and the PKN group at AF1 were separated from the others in the same group. Moreover, the samples of both groups at AF1 and both groups at AF21 respectively exhibited more similar viral community compositions (Figure 3b).

High throughput sequencing of 16S rRNA gene amplicons was performed to investigate bacterial diversity (general features of the high throughput sequencing results shown in Supplementary Materials Table S3). According to the Shannon index, the highest diversity of both groups was observed in the FP samples (11.67 in the PK group and 11.64 in the PKN group) (Figure 3a), but no significant difference was found between the two groups (*p* = 0.509) (Supplementary Materials Table S2). Irrigation resulted in the lower Shannon indices in both groups at FL3, and bacterial diversity in the PKN group was significantly higher than that in the PK group (*p* = 0.006). After the fertilizer application, the PK group had a higher Shannon index at AF1, and then got the lowest value at AF4 (Figure 3a). The urea application did not lead to evident changes in bacterial diversity at AF1 (*p* = 0.492), but Shannon indices exhibited higher at the next three sampling times. Bacterial diversity was significantly higher at AF1 in the PK group than in the PKN group (*p* = 0.018), but opposite results were observed at AF4 and AF14 (*p* = 0.000, *p* = 0.011, respectively). Shannon indices of both groups were higher at AF21 (11.48 in PK and 11.54 in PKN) than those at AF14, but there was no difference in Shannon indices between the two groups at AF21 (*p* = 0.064) (Supplementary Materials Table S2).

The UniFrac-coupled PCoA results illustrated the dynamics of bacterial communities on different sampling days. The bacterial communities of the PK and PKN groups were generally separated into two groups along axis 2, which expressed 21.6% of the variance in the unweighted UniFrac (Figure 3c). The largest distance of bacterial composition between the two groups was found at FP (Supplementary Materials Figure S2), and the FP results of both groups were separated along axis 1 explaining 46.8% of the variance (Figure 3c). However, the FP samples of both groups showed similar diversity indices. This indicated that long-term urea fertilization led to variation in the bacterial community structure.

In addition, based on 16S rRNA gene sequencing data, we investigated dominant bacterial populations at the phylum and genus levels. First, at the phylum level, there were no significant changes in community structure or relative abundance among the top 10 phyla. Proteobacteria, Acidobacteria, and Chloroflexi were the dominant populations at the phylum level in both groups (Supplementary Materials Figure S3). Second, at the genus level, *Nitrospira* was highly abundant in (relative abundance >1%) all samples in both groups except FP samples of the PKN group; *Bacillus* was highly abundant in all samples in both groups except AF14 samples of the PKN group; *Sphingomonas* was highly abundant in FP and AF4 samples in both groups (Supplementary Materials Figure S3, Table S4). Notably, in both groups, the dominant genera in the FP samples were almost the same as those in the flooded soils (*p* = 0.99 in PK, and *p* = 0.51 in PKN), which indicated that changes in SWC showed less effect on the composition of dominant bacterial populations.

### 3.3. Correlations between Viral Lytic Environment and Bacterial Community

The correlation analysis tested the relationship between VA, BA, VBR, microbial processes (CH_4_, CO_2_ and N_2_O), environmental factors (SOM, pH, available P, SWC, and soil temperature at 5 cm depth) (Supplementary Materials Table S5). There was no precipitation throughout the experimental period, therefore the effect of precipitation was not discussed. Before fertilization, VA in the PK group was positively correlated to VBR, SOM, available P, and CO_2_ emissions, negatively to BA, pH, SWC, and CH_4_ and N_2_O emissions. In the PKN group, VA was positively correlated with VBR and CO_2_ emissions, and was negatively correlated with SWC and N_2_O emissions. After applying fertilizer, in the PK group, VA was positively correlated to VBR and SOM, and negatively correlated to pH and available P. In the PKN group, VA was positively correlated with VBR and CH_4_ emissions, while negatively correlated with BA. Unexpectedly, although temperature is an important factor influencing viral survival [2], it was not correlated to VA in our results.

**Figure 4:**
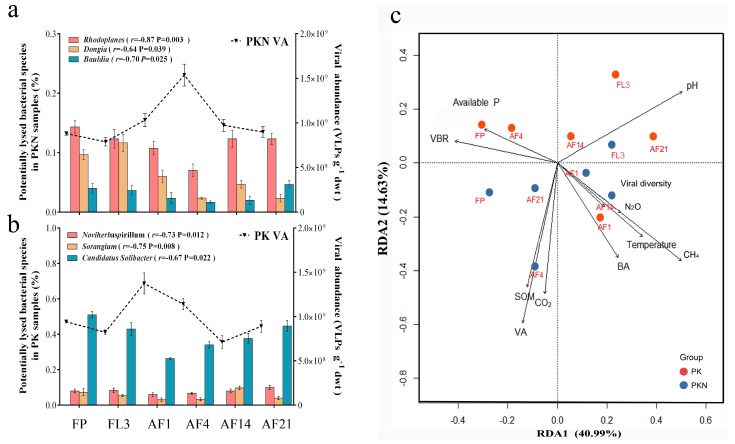
Bacterial community structure driven by potential specific viral predators and related factors. (**a**,**b**) The bar plots show the top three bacterial genera which most negatively correlated with the viral abundance (VA) over time (the correlation coefficient and significance are shown in the legends); (**c**) RDA analysis of all samples, indicating how was bacterial community driven by virus and other environment factors. The percentage of the variance explained by each axis is indicated in parenthesis. Red and blue filled circles indicate bacterial community of PK and PKN groups, respectively. VA, bacterial abundance (BA), VBR (virus-to-bacterium ratio), viral diversity index, microbial processes, and environmental variables are showed by arrows. Arrow length indicates the importance of a corresponding parameter; a small angle between two arrows indicates a strong correlation between the two corresponding parameters. Additionally, “Temperature” means soil temperature at 5 cm depth. “Viral diversity” means viral Shannon index. FP, representing spring fallow period; FL3, third day of flooding; AF1, 4, 14, 21 refer to the first, fourth, 14th, and 21st days after fertilization, respectively.

According to the dynamics of VA and the relative abundance of bacterial genera, those genera whose relative abundance were most significantly negatively correlated to the VA were selected as the potential lysed bacterial genera. They were *Rhodoplanes*, *Bauldia*, and *Dongia* in the PKN group and *Noviherbaspirillum*, *Sorangium*, and *Candidatus Solibacter* in the PK group (Figure 4a,b, respectively). The decrease in bacterial genera corresponded to an increase in VA, indicating that viral regulation in paddy soils is specifically targeted.

The contributions of variables were analyzed by RDA. Both RDA axes were significant (RDA1 *p* = 0.005, RDA2 *p* = 0.002), with axis 1 explaining 40.99% and axis 2 explaining 14.63% of the variation (Figure 4c). Soil pH, CH_4_ emissions, and VBR were identified as the most important predictors for axis 1; while for axis 2 the most important variables were VA, CO_2_ emissions, and SOM (Supplementary Materials Table S7). SWC was rejected by RDA because SWC was at an almost constant level after irrigation. Compared with that of the PKN group, microbial composition in the PK group was mainly driven by soil pH, available P, and VBR. In addition, VA was found to be significantly positively correlated with SOM. Meanwhile, VA, expressed with one of the longest arrows in the RDA analysis panel (Figure 4c), was an important influencing factor of microbial composition in the PKN group.

## 4. Discussion

Viruses are abundant and diverse, and thus are an important driving force of ecosystem functions [35]. By splitting specific subsets of microbiota, viruses determine the evenness and richness of microbial populations, and consequently affect microbial community structure [5,36]. Therefore, it is widely accepted that viruses play a pivotal role in biogeochemical nutrient and energy cycling [36]. Over the past two decades, research on viruses has mainly focused on aquatic ecosystems and little progress has been made in research on soil viruses in terrestrial ecosystems, especially regarding viral impact on their bacterial hosts, and consequently on nutrient cycling in agricultural ecosystems. In the present study, the dynamic changes in autochthonous soil viruses and their interactions with bacteria were studied in a long-term urea-treated paddy soil under the conditions of flooding and urea application, aiming to extend our knowledge of soil viruses in agricultural ecosystems.

### 4.1. Dynamic of VA, BA, and VBR

In this study, compared to the PK group, there were significantly lower soil viral and bacterial abundances in the fallow period under long-term (~30 years) urea application (Figure 2). Data also show that long-term urea fertilization resulted in a lower SOM in the PKN group during the fallow period (Table 1). The literature suggests that SOM has a positive effect on viral [20] and bacterial [19] abundances, which suggests that the decrease in VA and BA in the PKN group was the result of decreased SOM caused by long-term urea application. Additionally, extremely lower viral diversity was observed under the PKN treatment compared with that under the PK treatment at FP, whereas the bacterial diversity between the two groups was not significantly different (Figure 3a, Supplementary Materials Table S2). Since the lytic behavior of viruses has preferential targets [11], non-significant differences in bacterial diversity between the two groups at FP cannot explain the significant differences in viral diversity at FP. This indicated that bacterial lytic resistance may exist in paddy soils with long-term urea application, which blocks autochthonous viral reproduction and decreases viral diversity.

Viral diversity showed a significant increase in the PKN group after the third day of irrigation, which resulted in the similar viral diversity between two groups (Figure 3a). Meanwhile, viral abundance at FL3 also showed no difference between two groups (Supplementary Materials Table S1). Therefore, it is suggested that the dramatic increase in SWC led to a certain degree of convergence of soil viruses in the two groups. On the one hand, spatial heterogeneity (e.g., the simultaneous existence of water-filled pores and gas-filled pores blocks viral-bacterial interaction) [10,11] is an important factor in bacterial escape from phage infection in soil [24,37]. The soil in the PKN group at fallow period probably showed higher spatial heterogeneity than that in PK. After irrigation, the increase of SWC can bring about the improvement of soil spatial connectivity, thus promoting the viral mobility and infection rate. On the other hand, we observed a significant shift of soil bacterial community in the PKN group from fallow period to the third day after irrigation (Supplementary Materials Table S2). We speculate that the shift due to irrigation increases the population and variety of susceptible bacteria which benefit the replication of diverse viruses. Collectively, after irrigation, improved viral mobility, and increased population and variety of susceptible bacteria are the probable reasons that together result in a certain degree of convergence of soil viruses in the two groups at the third day of irrigation.

The increase in SWC did not induce variation in BA between the two groups at FL3, but Shannon indices of both treatments were significantly lower (Supplementary Materials Tables S1,2). Besides, the insignificant relationship between SWC and BA was also confirmed by the results of the correlation analysis (Supplementary Materials Table S7). This indicated that it was bacterial diversity, not abundance that SWC strongly regulated. Viruses can control the height of the fitness peaks of bacteria [38,39], and most viruses are host specific [11,29], thus the decrease of some targeted bacterial lineages can bring out the proliferation of other bacterial lineages, which leads to a maintained level of abundance and a varied diversity of bacteria. 

Concerning the VA results after short-term urea application (AF1), we found that the VA peak was delayed for three days in the PKN group compared with that in the PK group. Meanwhile, at AF1, a decrease in VBR was observed in the PKN group (Figure 2), indicating that viruses being temperate and switching from lysogenic to lytic cycle at this time spot (invalid infection and limitations of enumeration are also considerable reasons). All the above findings suggested that soil viruses undertake different reproduction strategies in the early stage after nitrogen fertilization. 

From the variation in VA and VBR between FL3 and AF1 in the PK group (Figure 2), we deduced that viruses rapidly respond to bacterial proliferation triggered by the addition of phosphate and potassium fertilizers, which shows a viral lytic incident. We suggested that this result is in accordance with “Kill-the-Winner” (KtW) model, in which virulent viruses regulate bacterial populations by eradicating the most abundant bacterial populations [10,40] (it is a suggestion because it cannot be absolutely confirmed when there is just count data). It was reported that dramatic viral infection under the KtW model led to an increase in VBR [10]. Therefore, the higher VBR in the PK group at AF1 than that in the PKN group also supports our speculation (Figure 2). In contrast to the PK group, low VBR after the first day of urea addition is the result of either resistant bacteria or temperate viruses that persist as proviruses from lysogenic infection, as reported in previous studies [11,29,36,41,42]. Moreover, a “Piggy-back-the-Winner” model (PtW) was recently described by Knowles et al. [8], in which, in a nutrient-rich environment, viruses may exploit their hosts through lysogeny rather than killing them. This model may explain lower VBR observed after the first day of urea application. Notably, the PtW model was first observed in a eutrophic aquatic ecosystem [8]; however, it was also reported that viruses follow the KtW model under conditions with sufficient carbon substrates in unsaturated soil [25]. Apparently, paddy soils can provide a completely different soil microenvironment for viruses than that in dry farming soil. A temporary eutrophic environment is created by urea addition (e.g., the PKN group at AF1) [43], and viral mobility is improved by high SWC [10]. Therefore, we propose that these are the probable reasons that viruses tend to transitorily exhibit a PtW pattern after urea application. 

VA in the PKN group peaked and exhibited its highest burst sizes at AF4, and was accompanied by an increase in VBR (Figure 2). This indicates that the PtW pattern observed at AF1 had switched to the KtW pattern at AF4. As urea is a kind of fast-effective fertilizer, we speculate that the reason for viruses changing their lifestyle strategy is that the brief eutrophication period has ceased by AF4. In addition, BA did not increase in both groups at AF4, and even significantly decreased in the PK group (*p* = 0.011) (Figure 2, Supplementary Materials Table S1), which suggests a top-down control of viruses. It also suggested that most observed viruses were related to bacteriophages. In addition, we found that bacterial diversity in the PK group decreased by 4.03% (*p* < 0.05) between AF1 and AF4, which accompanied with the viral diversity peak (Figure 3a,b, Supplementary Materials Table S2). This indicates a strong viral top-down control at the fourth day after fertilization. Whereas bacterial diversity in the PKN group showed a much smoother trend during the experimental period than that in the PK group. Further, bacterial diversity in the PKN group did not respond to the increase in viral diversity at AF4 (Figure 3a,b). We speculate that it is the result of increased resistance in the bacterial populations in urea-treated group.

By the final sampling day, BA peaked under both treatments, accompanied by a decrease in VA and the lowest VBR (Figure 2), which corresponded with previous results reported by Srinivasiah et al. [25]. After fertilizer application, in both groups, the increased growth rate in BA initially resulted in an increase in soil VA (Figure 2), and by the end, viral production had weakened relatively to viral decay. This indicates that the regulatory behavior of viruses weakens, or that resistance increases in the host populations [25].

### 4.2. Viral and Bacterial Community

RAPD-PCR is not a marker-gene based methodology for viral communities [33]; therefore, we cannot definitively demonstrate that the electrophoresis pattern completely characterizes the underlying changes in viral community structure. Nevertheless, the significant statistical test results prompt us to suggest that RAPD-PCR fingerprints can still provide credible results. Based on the subsequent PCA (Figure 3b), the great distance between the two groups during the spring fallow period, in the FP samples, demonstrates a strong driving force of long-term chemical-N fertilization (~30 years) on soil viral communities. Meanwhile, results showed that the viral composition of the PKN group at AF1 and the PK group at AF4 were more independent than the other samples among treatment-matched samples (Figure 3b). This indicates that irrigation and urea application have a strong driving effect on soil viral composition; however, viruses evidently respond more rapidly to urea application. In addition, at the end of the experimental period, urea addition did not drive the PKN group to form a different viral community than that in the PK group (Figure 3b), From the results of PERMANOVA, values in the two groups have no significant difference (Supplementary Materials Table S2), and both viral Shannon indices and VBR reach their lowest values (Figure 3a, 2). This indicates a weakening of viral activity at the end of the experimental period.

From the results of PERMANOVA, dominant bacterial genera (>1%) were roughly the same between two groups (Supplementary Materials Table S6). However, from examining the sequencing data, PCoA results also show two clusters of generally separated bacterial community positions under the treatment groups (Figure 3c). This suggests that long-term urea application mostly affects non-dominant bacterial populations, and thus modifies bacterial composition. Meanwhile, RDA showed that the cluster of the microbes in the PKN group was strongly driven by VA (Figure 4c), which demonstrates that the dynamics of VA are linked to a variation in bacterial communities. As previously reported, nutritional changes caused by urea addition are one of the main drivers of microbial shifts in soil systems [44,45]. Additionally, in the PK group, which had added phosphorus and potassium, available phosphorus was shown to be an important factor for bacterial community composition. This suggests once again that long-term fertilization has a vital effect on bacterial communities. Notably, despite the lower soil moisture before irrigation, the dominant genera in the FP samples were almost the same as those in the flooded soils. This indicates that a dramatic change in SWC by flooding did not change the dominant genera present. Undiversified dynamics of dominant genera was also probably due to microaerophilic habitat in irrigated soil (especially some microaerophilic pore spaces that sheltering aerobic bacteria), which maintained the abundance of dominant bacterial genera. Contamination from relic DNA [46,47] also should be the considerable reason.

In this study, potential lysed bacterial genera were determined by the correlation analysis between the VA and relative abundance of bacterial genera. Methodologically, an increased VA and a decreased BA could mean the bacteria are lysed. However, in our study, VA data were obtained by applying counting methods, and relative abundances of bacterial genera were collected from sequencing data. Methodological inconsistencies may lead to some uncertainties that promote us to use “potential” for rigor [48]. It has been reported that viruses rapidly respond to changes in the abundance of specific bacterial host populations [11]. Similarly, our results showed that bacteriophage regulation of bacteria was not only limited to bacterial composition. The observed trade-off between VA and potential lysed genera indicates that virus regulation in paddy soils has specific targets (Figure 4a,b). In the PKN group, the potential lysed genera of *Rhodoplanes* and *Dongia* were reported as nitrogen-fixing bacteria [49,50]. *Bauldia* is the order *Rhizobiales* which also has the ability of nitrogen fixation [51,52]. That is to say, in the PKN group, that nitrogen-fixing populations were lysed. This indicates that bacteriophages kill nitrogen-fixing bacteria, and thus limit their ability to absorb nitrogen from the air, thereby reducing excessive nitrogen accumulation. While in the PK group, two of the most lysed bacteria, *Noviherbaspirillum* and *Sorangium* function in denitrification [53,54], suggesting that bacteriophages decrease the loss of soil nitrogen to the air. In addition, potential lysed bacterial genera in the PKN group (*Rhodoplanes, Dongia*, and *Bauldia*) were not correlated to VA in the PK group (Supplementary Materials Figure S5). This confirmed that the specificity of bacteriophages lysis is induced by urea application. Overall, the above results imply that the specifically lysed bacterial populations are closely related to soil nutrient cycling.

A previous study showed that the type of N fertilizer affects the evolution of N_2_, N_2_O, and CH_4_ from a flooded-rice soil to the atmosphere [55], which are indicators of soil N and C cycling. In our study, we observed a higher CH_4_ emission rate in the urea-treated group (Supplementary Materials Figure S4), which suggests that a correlation may exist between urea addition and CH_4_ producers. Coincidentally, the abundance of *Methanosarcina*, one of the chief CH_4_ producing archaea, was positively correlated to VA only in the PKN group (Supplementary Materials Figure S5). As we expected, CH_4_ emissions were only positively correlated to VA and VBR after applying urea (Supplementary Materials Table S5). Therefore, we deduced that soil viruses should be involved in the regulation of CH_4_ production under urea-treated conditions. In addition, the results of previous research suggest that *Methanosarcina* may be the only anaerobic methanogen that produces methane using all three known methanogenic metabolic pathways, and that it can use more than nine methanogenic substrates, including CO_3_^2−^ hydrolyzed by urea [56]. From this understanding, we consider it possible that methanogens rapidly metabolize CO_3_^2−^ produced from urea hydrolysis and enhance CH_4_ production. In our opinion, these are reasonable assumptions for how CH_4_ production responds to VA under urea addition, which can quickly balance soil nutrients in this agricultural ecosystem. As far as we know, this is the first report on the influence of soil viruses and CH_4_ emissions.

## 5. Conclusions

Nutrient cycling in agricultural ecosystems is a complicated problem. Most previous reports were based on the driving role of environmental factors. With the progress of molecular biology techniques, the role of soil microorganisms in nutrient cycling has been focused on more. However, the role of soil viruses is seldom considered in studies. Our study advances an understanding of the role of autochthonous soil viruses in a long-term urea-treated paddy soil. First, soil viruses respond rapidly to urea addition, which modifies the bacterial community composition and functions. Second, in paddy soil, viruses show diversified survival strategies under different soil conditions. Soil transient eutrophication caused by urea addition enables viruses to switch to temperate strategies in the short term. Subsequently, viruses become more virulent, which causes a variation in BA and community structure. Third, in response to the addition of fertilizers, viruses selectively lyse bacterial populations with specific functions, such as N cycling groups, thus regulating soil nutrients. Together, these results emphasize the crucial role of autochthonous soil viruses in nutrient cycling through the regulation of soil bacterial communities. Notably, to the best of our knowledge, this is the first report on the dynamics of autochthonous soil viruses in paddy soils.

## Figures and Tables

**Table 1:** Physicochemical properties^1^ of two treatments of soil.

Soil properties	PK	PKN
SOM^2^ (g/kg dw)	14.62	13.86
Total N (g/kg dw)	0.84	0.78
Total P (g/kg dw)	0.74	0.63
Available P (mg/kg dw)	18.62	12.53
Total K (g/kg dw)	19.75	19.93
Available K (mg/kg dw)	209.64	218.53
pH	6.86	7.15
SWC^3^ (%)	11.40	11.48

^1^ All of physicochemical properties in Table 1 represent the condition of the below mentioned sampling time “FP”; ^2^ SOM is the abbreviation of soil organic matter; ^3^ SWC represents soil mass water content.

## Supplementary Materials

The following are available online at https://www.mdpi.com/1999-4915/11/4/347/s1, **Figure S1**: RAPD electrophoretogram of viral diversity and cluster analysis of all samples. FP, representing spring fallow period; FL3, third day of flooding; AF1, 4, 14, 21 refer to the first, fourth, 14th, and 21st days after fertilization, respectively; **Figure S2**: UniFrac distance between bacterial communities on six sampling days. FP, representing spring fallow period; FL3, third day of flooding; AF1, 4, 14, 21 refer to the first, fourth, 14th, and 21st days after fertilization, respectively; **Figure S3**: Dominant bacterial populations and their composition: Top 10 phyla (a) and top 20 genera (b) of all samples. FP, representing spring fallow period; FL3, third day of flooding; AF1, 4, 14, 21 refer to the first, fourth, 14th, and 21st days after fertilization, respectively; **Figure S4**: Dynamics of CH_4_, CO_2_ and N_2_O emissions of all samples. FP, representing spring fallow period; FL3, third day of flooding; AF1, 4, 14, 21 refer to the first, fourth, 14th, and 21st days after fertilization, respectively; **Figure S5**: In the PK group, dynamics of bacteria which are potentially lysed in the PKN group; and in the PKN group, dynamics of bacteria which in the PK group are potentially lysed. FP, representing spring fallow period; FL3, third day of flooding; AF1, 4, 14, 21 refer to the first, fourth, 14th, and 21st days after fertilization, respectively; **Table S1**: Viral and bacterial abundance (×10^6^) and viral-to-bacterial ratio; **Table S2**: Viral and bacterial Shannon indices; **Table S3**: General features of the high throughput sequencing results and bacterial richness values; **Table S4**: Relative abundance (%) of top 20 bacterial genera of all samples; **Table S5**: Correlation analysis of viral and bacterial assemblages, and environmental factors; **Table S6**: PERMANOVA indicating no significances are observed in the composition of dominant bacterial genera (>1%) among all samples; **Table S7**: Accumulated constrained eigenvalues and biplot scores for constraining variables of RDA.

## References

[1] Rohwer F., Prangishvili D., Lindell D. (2009). Roles of viruses in the environment. Environ. Microbiol..

[2] Kuzyakov Y., Mason-Jones K. (2018). Viruses in soil: Nano-scale undead drivers of microbial life, biogeochemical turnover and ecosystem functions. Soil Biol. Biochem..

[3] Lindell D., Jaffe J.D., Coleman M.L., Futschik M.E., Axmann I.M., Rector T., Kettler G., Sullivan M.B., Steen R., Hess W.R. (2007). Genome-wide expression dynamics of a marine virus and host reveal features of co-evolution. Nature.

[4] Tuomi P., Fagerbakke K.M., Bratbak G., Heldal M. (1995). Nutritional enrichment of a microbial community: The effects on activity, elemental composition, community structure and virus production.pdf. FEMS Microbiol. Ecol..

[5] Wommack K.E., Colwell R.R. (2000). Virioplankton: Viruses in Aquatic ecosystems. Microbiol. Mol. Biol. Rev..

[6] Jover L.F., Effler T.C., Buchan A., Wilhelm S.W., Weitz J.S. (2014). The elemental composition of virus particles: implications for marine biogeochemical cycles. Nat. Rev. Microbiol..

[7] Maurice C.F., Bouvier C., de Wit R., Bouvier T. (2013). Linking the lytic and lysogenic bacteriophage cycles to environmental conditions, host physiology and their variability in coastal lagoons. Environ. Microbiol..

[8] Knowles B., Silveira C.B., Bailey B.A., Barott K., Cantu V.A., Cobian-Guemes A.G., Coutinho F.H., Dinsdale E.A., Felts B., Furby K.A. (2016). Lytic to temperate switching of viral communities. Nature.

[9] Liang X., Zhuang J., Löffler F.E., Zhang Y., DeBruyn J.M., Wilhelm S.W., Schaeffer S.M., Radosevich M. (2019). Viral and bacterial community responses to stimulated Fe(III)-bioreduction during simulated subsurface bioremediation. Environ. Microbiol..

[10] Pratama A.A., van Elsas J.D. (2018). The ‘neglected’ soil virome—potential role and impact. Trends Microbiol..

[11] Williamson K.E., Fuhrmann J.J., Wommack K.E., Radosevich M. (2017). Viruses in Soil Ecosystems: An Unknown Quantity Within an Unexplored Territory. Annu. Rev. Virol..

[12] Zablocki O., Adriaenssens E.M., Cowan D., Löffler F.E. (2016). Diversity and ecology of Viruses in hyperarid desert soils. Appl. Environ. Microbiol..

[13] Segobola J., Adriaenssens E., Tsekoa T., Rashamuse K., Cowan D. (2018). Exploring viral diversity in a unique South African soil habitat. Sci. Rep..

[14] Emerson J.B., Roux S., Brum J.R., Bolduc B., Woodcroft B.J., Jang H.B., Singleton C.M., Solden L.M., Naas A.E., Boyd J.A. (2018). Host-linked soil viral ecology along a permafrost thaw gradient. Nat. Microbiol..

[15] Trubl G., Jang H.B., Roux S., Emerson J.B., Solonenko N., Vik D.R., Solden L., Ellenbogen J., Runyon A.T., Bolduc B. (2018). Soil Viruses are underexplored players in ecosystem carbon processing. mSystems.

[16] Adriaenssens E.M., Kramer R., Van Goethem M.W., Makhalanyane T.P., Hogg I., Cowan D.A. (2017). Environmental drivers of viral community composition in Antarctic soils identified by viromics. Microbiome.

[17] Zablocki O., van Zyl L., Adriaenssens E.M., Rubagotti E., Tuffin M., Cary S.C., Cowan D. (2014). High-Level diversity of tailed phages, eukaryote-associated viruses, and virophage-like elements in the metaviromes of Antarctic soils. Appl. Environ. Microbiol..

[18] Wang X., Fan J., Xing Y., Xu G., Wang H., Deng J., Wang Y., Zhang F., Li P., Li Z. (2019). The Effects of mulch and Nitrogen fertilizer on the soil environment of crop plants. Adv. Agron..

[19] Chen L., Xun W., Sun L., Zhang N., Shen Q., Zhang R. (2014). Effect of different long-term fertilization regimes on the viral community in an agricultural soil of Southern China. Eur. J. Soil Biol..

[20] Williamson K.E., Radosevich M., Wommack K.E. (2005). Abundance and diversity of viruses in six delaware soils. Appl. Environ. Microbiol..

[21] Swanson M.M., Fraser G., Daniell T.J., Torrance L., Gregory P.J., Taliansky M. (2009). Viruses in soils: morphological diversity and abundance in the rhizosphere. An. Appl. Biol..

[22] Zhang Y., Liao J., Li F., Huang Y., Hu R., Yuan Z. (2002). Fixed ammonium content of chief paddy soil types in Hunan Province and its influencing factors. Chin. J. Appl. Ecol..

[23] Wang A., Su Y., Li Y., Hu L., Wu J. (2012). Response of the turnover of soil organic carbon to the soil moisture in paddy and upland Soil. Sci. Agric. Sin..

[24] Kimura M., Jia Z.-J., Nakayama N., Asakawa S. (2008). Ecology of viruses in soils: Past, present and future perspectives. Soil Sci. Plant Nutr..

[25] Srinivasiah S., Lovett J., Ghosh D., Roy K., Fuhrmann J.J., Radosevich M., Wommack K.E. (2015). Dynamics of autochthonous soil viral communities parallels dynamics of host communities under nutrient stimulation. FEMS Microbiol. Ecol..

[26] Dong D., Kou Y., Yang W., Chen G., Xu H. (2018). Effects of urease and nitrification inhibitors on nitrous oxide emissions and nitrifying/denitrifying microbial communities in a rainfed maize soil: A 6-year field observation. Soil Tillage Res..

[27] Lu R. (1999). Analysis of Soil Agrochemistry.

[28] Williamson K.E., Wommack K.E., Radosevich M. (2003). Sampling Natural viral communities from soil for culture-independent analyses. Appl. Environ. Microbiol..

[29] Williamson K.E., Radosevich M., Smith D.W., Wommack K.E. (2007). Incidence of lysogeny within temperate and extreme soil environments. Environ. Microbiol..

[30] Patel A., Noble R.T., Steele J.A., Schwalbach M.S., Hewson I., Fuhrman J.A. (2007). Virus and prokaryote enumeration from planktonic aquatic environments by epifluorescence microscopy with SYBR Green I. Nat. Protoc..

[31] Caporaso J.G., Kuczynski J., Stombaugh J., Bittinger K., Bushman F.D., Costello E.K., Fierer N., Peña A.G., Goodrich J.K., Gordon J.I. (2010). QIIME allows analysis of high-throughput community sequencing data. Nat. Methods.

[32] Edwards J., Johnson C., Santos-Medellin C., Lurie E., Podishetty N.K., Bhatnagar S., Eisen J.A., Sundaresan V. (2015). Structure, variation, and assembly of the root-associated microbiomes of rice. Proc. Nat. Acad. Sci. USA.

[33] Srinivasiah S., Lovett J., Polson S., Bhavsar J., Ghosh D., Roy K., Fuhrmann J.J., Radosevich M., Wommack K.E. (2013). Direct assessment of viral diversity in soils by random PCR amplification of polymorphic DNA.pdf. Appl. Environ. Microbiol..

[34] McMurdie P.J., Holmes S. (2013). Phyloseq: An R package for reproducible interactive analysis and Graphics of microbiome census data. PLoS ONE.

[35] Cobian Guemes A.G., Youle M., Cantu V.A., Felts B., Nulton J., Rohwer F. (2016). Viruses as winners in the game of life. Annu. Rev. Virol..

[36] Suttle C.A. (2007). Marine viruses—major players in the global ecosystem. Nat. Rev. Microbiol..

[37] Burroughs N.J., Marsh P., Wellington E.M.H. (2000). Mathematical analysis of growth and interaction dynamics of streptomycetes and a bacteriophage in soil. Appl. Environ. Microbiol..

[38] Beckett Stephen J., Williams Hywel T.P. (2013). Coevolutionary diversification creates nested-modular structure in phage–bacteria interaction networks. Interface Focus.

[39] Weitz J.S. (2015). Quantitative Viral Ecology: Dynamics of Viruses and Their Microbial Hosts.

[40] Korytowski D.A., Smith H.L. (2017). Permanence and Stability of a kill the winner model in marine ecology. Bull. Math. Biol..

[41] Kadavy D.R., Shaffer J.J., Lott S.E., Wolf T.A., Bolton C.E., Gallimore W.H., Martin E.L., Nickerson K.W., Kokjohn T.A. (2000). Influence of infected cell growth state on bacteriophage reactivation levels. Appl. Environ. Microbiol..

[42] Liang X., Radosevich M. (2019). Commentary: A host-produced quorum-sensing autoinducer controls a phage lysis-lysogeny decision. Front. Microbiol..

[43] Hamilton J.T.G., McRoberts W.C., Keppler F., Kalin R.M., Harper D.B. (2003). Chloride methylation by plant pectin: An efficient environmentally significant process. Science.

[44] Ramirez K.S., Lauber C.L., Knight R., Bradford M.A., Fierer N. (2010). Consistent effects of nitrogen fertilization on soil bacterial communities in contrasting systems. Ecology.

[45] Zeng J., Liu X., Song L., Lin X., Zhang H., Shen C., Chu H. (2016). Nitrogen fertilization directly affects soil bacterial diversity and indirectly affects bacterial community composition. Soil Biol. Biochem..

[46] Emerson J.B., Adams R.I., Román C.M.B., Brooks B., Coil D.A., Dahlhausen K., Ganz H.H., Hartmann E.M., Hsu T., Justice N.B. (2017). Schrödinger’s microbes: Tools for distinguishing the living from the dead in microbial ecosystems. Microbiome.

[47] Carini P., Marsden P.J., Leff J.W., Morgan E.E., Strickland M.S., Fierer N. (2016). Relic DNA is abundant in soil and obscures estimates of soil microbial diversity. Nat. Microbiol..

[48] Wang J., Gao Y., Zhao F. (2016). Phage–bacteria interaction network in human oral microbiome. Environ. Microbiol..

[49] Harada N., Nishiyama M., Otsuka S., Matsumoto S. (2005). Effects of inoculation of phototrophic purple bacteria on grain yield of rice and nitrogenase activity of paddy soil in a pot experiment. Soil Sci. Plant Nutr..

[50] Sun M., Xiao T., Ning Z., Xiao E., Sun W. (2015). Microbial community analysis in rice paddy soils irrigated by acid mine drainage contaminated water. Appl. Microbiol. Biotechnol..

[51] Kumar P.A., Srinivas T.N.R., Manasa P., Madhu S., Shivaji S. (2012). Lutibaculum baratangense gen. nov., sp. nov., a proteobacterium isolated from a mud volcano. Int. J. Syst. Evol. Microbiol..

[52] Black M., Moolhuijzen P., Chapman B., Barrero R., Howieson J., Hungria M., Bellgard M. (2012). The Genetics of symbiotic nitrogen fixation: Comparative genomics of 14 rhizobia strains by resolution of Protein clusters. Genes.

[53] Qian W., Ma B., Li X., Zhang Q., Peng Y. (2019). Long-term effect of pH on denitrification: High pH benefits achieving partial-denitrification. Biores. Technol..

[54] Oshiki M., Segawa T., Ishii S. (2018). Nitrogen cycle evaluation (nice) chip for simultaneous analysis of multiple n cycle-associated genes. Appl. Environ. Microbiol..

[55] Lindau C.W., DeLaune R.D., Patrick W.H., Bollich P.K. (1990). Fertilizer effects on dinitrogen, nitrous oxide, and methane emissions from lowland rice. Soil Sci. Soc. Am. J..

[56] Galagan J.E., Nusbaum C., Roy A., Endrizzi M.G., Macdonald P., FitzHugh W., Calvo S., Engels R., Smirnov S., Atnoor D. (2002). The genome of M. acetivorans reveals extensive metabolic and physiological diversity. Genome Res..

