# Supplementary material for: Dynamics of Bacterial and Viral Communities in Paddy Soil with Irrigation and Urea Application
Source: Viruses. 2019 Apr 16;11(4):347. doi: 10.3390/v11040347 (PMC6520780; doi:10.3390/v11040347)
Supplement: Supplementary file 1 [file viruses-11-00347-s001.pdf]

Dynamics of bacterial and viral communities in paddy soil with irrigation and urea application

Yuting Li [1,2,3], Hao Sun [1,4], Weichao Yang [1,4], Guanxiong Chen [1] and Hui Xu [1,4,] *

1 Institute of Applied Ecology, Chinese Academy of Sciences, Shenyang 110016, China; liyuting211@mails.ucas.ac.cn (Y.L.); haos@spaces.ac.cn (H.S.); yangweichao@iae.ac.cn (W.Y.); gxchen39@iae.ac.cn (G.C.)

2 University of Chinese Academy of Sciences, Beijing 100049, China

3 National Field Observation and Research Station of Shenyang Agro-Ecosystems, Shenyang 110016, China

4 Key Laboratory of Pollution Ecology and Environmental Engineering, Institute of Applied Ecology, Chinese Academy of Sciences, Shenyang 110016, China

* Correspondence: xuhui@iae.ac.cn (H.X.); Tel.: +86-024-8397-0369

Supporting Information

Supplementary Table S1 Viral and bacterial abundance (×10^6) and viral-to-bacterial ratio.

	Viral abundance		Bacterial abundance		Virus / bacteria ratio	
	PK	PKN	PK	PKN	PK	PKN
FP	942.87±15.33a	881.48±38.46b	3.17±0.28ae	2.49±0.33d	300.23±31.85ae	362.53±58.47e
FL3	822.79±53.34b	788.29±94.46b	3.53±0.28a	2.65±0.37de	234.91±30.00b	302.07±47.76e
AF1	1374.01±236.38cd	1033.13±148.19ac	5.67±0.42b	6.42±0.24f	241.77±34.20b	161.63±26.27c
AF4	1141.85±122.61c	1537.05±247.20d	4.45±0.61c	6.17±0.56bf	261.69±43.57ab	248.37±27.18b
AF14	711.71±148.28b	972.58±132.71ab	5.95±0.49bf	8.48±1.08g	120.49±26.53cf	116.77±24.17f
AF21	889.82±137.06ab	899.36±119.26ab	6.04±1.51bcfg	8.71±1.86g	153.06±28.58cf	107.28±22.45fg

Different letters within a column show significant differences ($p < 0.05$)

FP, representing spring fallow period; FL3, third day of flooding; AF1, 4, 14, 21 refer to the first, fourth, 14th, and 21st days after fertilization, respectively.

Supplementary Table S2 Viral and bacterial Shannon indices.

	Viral Shannon index		Bacterial Shannon index	
	PK	PKN	PK	PKN
FP	2.84±0.07a	2.01±0.02d	11.67±0.07a	11.64±0.06af
FL3	2.97±0.15ace	3.01±0.02e	11.24±0.02b	11.31±0.01e
AF1	2.81±0.09a	3.04±0.05e	11.41±0.05c	11.31±0.01e
AF4	3.51±0.05b	3.35±0.03f	10.95±0.06d	11.36±0.05ce
AF14	3.02±0.06c	3.09±0.13c	11.33±0.02e	11.41±0.03c
AF21	2.03±0.06d	2.01±0.02d	11.48±0.01cf	11.54±0.04f

Different letters within a column show significant differences ($p < 0.05$)

FP, representing spring fallow period; FL3, third day of flooding; AF1, 4, 14, 21 refer to the first, fourth, 14th, and 21st days after fertilization, respectively.

Supplementary Table S3 General features of the high throughput sequencing results and bacterial richness values.

	Total reads		Observed OTUs		Chao1 index	
	PK	PKN	PK	PKN	PK	PKN
FP	112315±23631	92633±28849	5499±150	5371±97	18765±1080	17811± 1200
FL3	76935±20102	108327±35054	4806±27	4942±102	15145±838	15086± 559
AF1	126941±27066	141728±16901	5078±99	4987±45	16579±531	15069± 179
AF4	113166±46749	140313±88110	4596±53	4966±75	13989±1290	16443± 180
AF14	92892±27798	125079±52420	4950±44	5148±47	16426±171	16144± 556
AF21	103746±22575	134966±48337	5192±33	5297±40	16734±265	16810± 774

FP, representing spring fallow period; FL3, third day of flooding; AF1, 4, 14, 21 refer to the first, fourth, 14th, and 21st days after fertilization, respectively.

Supplementary Table S4 Relative abundance (%) of top 20 bacterial genera of all samples.

	FP		FL3		AF1		AF4		AF14		AF21	
	PK	PKN	PK	PKN	PK	PKN	PK	PKN	PK	PKN	PK	PKN
Nitrospira	1.357	1.327	2.563	2.827	2.347	2.270	2.043	1.807	2.563	2.213	2.870	1.390
Bacillus	2.027	0.930	1.220	2.270	1.700	3.253	3.717	1.103	1.767	0.840	1.603	1.700
Sphingomonas	1.117	1.610	0.717	0.637	0.850	0.567	1.097	1.250	0.540	0.490	0.490	0.867
Geobacter	0.690	0.657	3.070	0.243	0.490	0.270	0.253	0.253	0.547	0.717	0.987	1.243
Aquicella	0.390	0.633	0.593	0.827	0.910	0.813	0.263	0.567	0.710	1.490	0.807	0.250
OM43 clade	0.067	0.027	1.653	0.537	0.703	0.023	2.903	0.817	0.757	0.113	0.110	0.030
Haliangium	0.593	0.413	0.693	0.580	0.620	0.630	0.477	0.360	0.403	0.397	0.540	0.600
Opitutus	0.153	0.103	0.537	0.623	0.580	0.967	0.173	0.520	0.607	1.013	0.600	0.393
Ferruginibacter	0.570	0.763	0.570	0.323	0.493	0.557	0.560	0.777	0.263	0.377	0.477	0.287
Arthrobacter	1.280	0.533	0.443	0.293	0.353	0.263	0.420	0.307	0.583	0.213	0.420	0.620
Pseudomonas	0.417	0.163	0.793	0.333	0.397	0.660	0.430	0.433	0.230	0.400	0.437	0.977
Lactococcus	0.237	0.087	0.280	0.573	0.510	0.777	1.210	0.307	0.483	0.157	0.463	0.490
Blastocatella	0.490	0.997	0.370	0.183	0.417	0.283	0.423	0.753	0.350	0.407	0.380	0.343
Candidatus Solibacter	0.510	0.743	0.430	0.287	0.263	0.437	0.340	0.387	0.377	0.453	0.447	0.553
Clostridium sensu stricto 1	0.360	1.100	0.397	0.707	0.133	0.597	0.133	0.450	0.097	0.747	0.217	0.207
Candidatus Nitrosopumilus	0.060	0.017	0.510	0.697	0.990	0.777	0.040	0.037	0.247	0.790	0.590	0.367
Bryobacter	0.697	0.907	0.363	0.240	0.377	0.283	0.293	0.350	0.257	0.343	0.330	0.377
Roseiflexus	0.510	0.507	0.387	0.493	0.303	0.290	0.317	0.480	0.527	0.300	0.287	0.403
Longilinea	0.393	0.893	0.290	0.327	0.310	0.357	0.247	0.493	0.147	0.423	0.280	0.353
Flavobacterium	0.147	0.130	0.633	0.523	0.133	0.283	0.117	0.660	0.133	0.207	0.283	0.883

FP, representing spring fallow period; FL3, third day of flooding; AF1, 4, 14, 21 refer to the first, fourth, 14th, and 21st days after fertilization, respectively.

Supplementary Table S5 Correlation analysis of viral and bacterial assemblages, and environmental factors.

	VA	BA	VBR
**CK group, before fertilization**			
VA	1	-0.709 [0.022]*	0.930 [0.000]*
BA	-0.709 [0.022]*	1	-0.894 [0.000]*
VBR	0.930 [0.000]*	-0.894 [0.000]*	1
pH	-0.756 [0.011]*	0.543 [0.105]	-0.673 [0.033]*
SWC	-0.867 [0.001]*	0.394 [0.260]	-0.687 [0.028]*
SOM	0.794 [0.006]*	-0.430 [0.214]	0.638 [0.047]*
Available P	0.806 [0.005]*	-0.406 [0.244]	0.669 [0.035]*
Temp	0.079 [0.828]	0.395 [0.258]	-0.104 [0.776]
CH_4 emission	-0.745 [0.013]*	0.418 [0.229]	-0.596 [0.069]
CO_2 emission	0.879 [0.001]*	-0.685 [0.029]*	0.809 [0.005]*
N_2O emission	-0.794 [0.006]*	0.721 [0.019]*	-0.772 [0.009]*
**CK group, after fertilization**			
VA	1	-0.168 [0.478]	0.886 [0.000]*
BA	-0.168 [0.478]	1	-0.564 [0.010]*
VBR	0.886 [0.000]*	-0.564 [0.010]*	1
pH	-0.700 [0.001]*	0.166 [0.485]	-0.627 [0.003]*
SWC	0.415 [0.069]	0.038 [0.872]	0.308 [0.187]
SOM	0.553 [0.011]*	0.093 [0.696]	0.408 [0.075]
Available P	-0.526 [0.017]*	0.463 [0.040]*	-0.687 [0.001]*
Temp	-0.005 [0.985]	-0.124 [0.602]	0.033 [0.890]
CH_4 emission	0.074 [0.758]	-0.002 [0.995]	0.081 [0.734]
CO_2 emission	-0.344 [0.137]	0.598 [0.005]*	-0.517 [0.020]*
N_2O emission	-0.075 [0.753]	0.162 [0.494]	-0.033[0.890]
**U group, before fertilization**			
VA	1	-0.224 [0.533]	0.809 [0.005]*
BA	-0.224 [0.533]	1	-0.657 [0.039]*
VBR	0.809 [0.005]*	-0.657 [0.039]*	1
pH	-0.444 [0.199]	-0.146 [0.688]	-0.329 [0.353]
SWC	-0.697 [0.025]*	0.467 [0.174]	-0.809 [0.005]*
SOM	0.079 [0.829]	0.152 [0.676]	0.176 [0.626]
Available P	0.430 [0.214]	-0.030 [0.934]	0.340 [0.336]
Temp	0.383 [0.275]	0.432 [0.213]	0.055 [0.880]
CH_4 emission	-0.297 [0.405]	0.224 [0.533]	-0.365 [0.300]
CO_2 emission	0.661 [0.038]*	-0.503 [0.138]	0.821 [0.004]*
N_2O emission	-0.685 [0.029]*	0.273 [0.446]	-0.644 [0.044]*
**U group, after fertilization**			
VA	1	-0.499 [0.025]*	0.845 [0.000]*
BA	-0.499 [0.025]*	1	-0.835 [0.000]*
VBR	0.845 [0.000]*	-0.835 [0.000]*	1
pH	-0.429 [0.053]	0.477 [0.033]*	-0.600 [0.005]*
SWC	0.199 [0.401]	-0.370 [0.108]	0.364 [0.114]
SOM	0.021 [0.930]	0.245 [0.298]	-0.050 [0.835]
Available P	0.170 [0.474]	-0.149 [0.531]	0.216 [0.361]
Temp	0.073 [0.760]	0.126 [0.598]	0.051 [0.830]
CH_4 emission	0.415 [0.048]*	-0.403 [0.078]	0.453 [0.045]*
CO_2 emission	-0.101 [0.673]	0.026 [0.915]	-0.084 [0.724]
N_2O emission	-0.168 [0.478]	0.296 [0.205]	-0.227 [0.336]

VA, viral abundance; BA, bacterial abundance; VBR, virus-to-bacterium ratio; SOM, soil organic matter; SWC, soil water content

*Significant correlations ($P \leq 0.05$). Probability values in square brackets.

Supplementary Table S6 PERMANOVA indicating no significances are observed in the composition of dominant bacterial genera (>1%) among all samples.

	Df	Sums Of Sqs	Mean Sqs	F. Model	R2	Pr(>F)	Sig.
**Group**	1	0.03585	0.035851	1.3905	0.03929	0.246	ns
Residuals	34	0.87661	0.025783	0.96071			
Total	35	0.91246	1				

Supplementary Table S7 Accumulated constrained eigenvalues and biplot scores for constraining variables of RDA.

Accumulated constrained eigenvalues

	Eigenvalue	Proportion explained	Cumulative proportion
RDA1	0.004882	0.409856	0.409856
RDA2	0.001743	0.146285	0.556141
RDA3	0.001489	0.124976	0.681118
RDA4	0.001206	0.101272	0.78239
RDA5	0.001101	0.092444	0.874834
RDA6	0.000622	0.052171	0.927004
RDA7	0.000341	0.028643	0.955647
RDA8	0.000263	0.022035	0.977682
RDA9	0.000117	0.009812	0.987494
RDA10	8.06E-05	6.77E-03	9.94E-01
RDA11	6.84E-05	5.74E-03	1.00E+00

Biplot scores for constraining variables

	RDA1	RDA2	RDA3	RDA4	RDA5	RDA6
VA	-0.17401	-0.7395	0.05952	0.12979	-0.3613	0.1815
BA	0.30586	-0.4369	0.1955	0.05198	0.2219	0.5946
VBR	-0.51331	0.1016	-0.34365	-0.05486	-0.2807	-0.4592
Available_P	-0.36655	0.1564	0.35427	-0.11281	0.6758	-0.131
pH	0.62556	0.329	0.14503	-0.26766	-0.3094	0.1331
Viral Diversity	0.23533	-0.2019	0.26924	0.36752	-0.3269	-0.3342
SOM	-0.15275	-0.574	-0.46407	0.11944	0.1038	0.3481
Temperature	0.4268	-0.3398	0.13022	0.29123	0.2542	0.3852
CH_4	0.62193	-0.451	0.19795	0.27213	-0.3633	0.306
CO_2	-0.06302	-0.6043	0.09608	-0.12022	0.1455	0.2487
N_2O	0.32207	-0.2334	-0.1596	-0.26671	0.028	0.1813

VA, viral abundance; BA, bacterial abundance; VBR, virus-to-bacterium ratio; SOM, soil organic matter

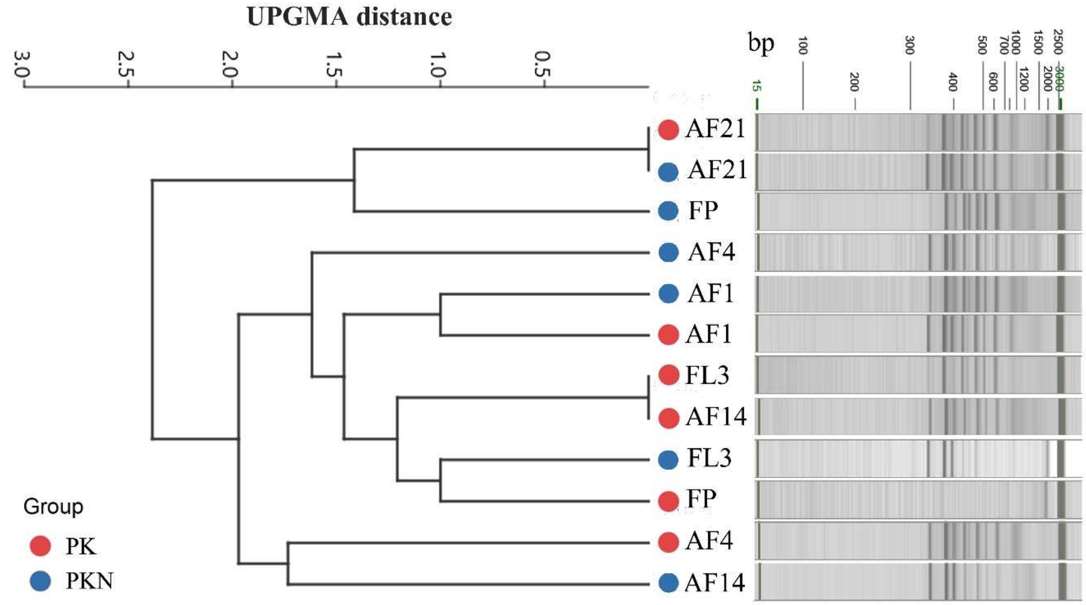

Supplementary Figure S1 RAPD electrophoretogram of viral diversity and cluster analysis of all samples. FP, representing spring fallow period; FL3, third day of flooding; AF1, 4, 14, 21 refer to the first, fourth, 14th, and 21st days after fertilization, respectively.

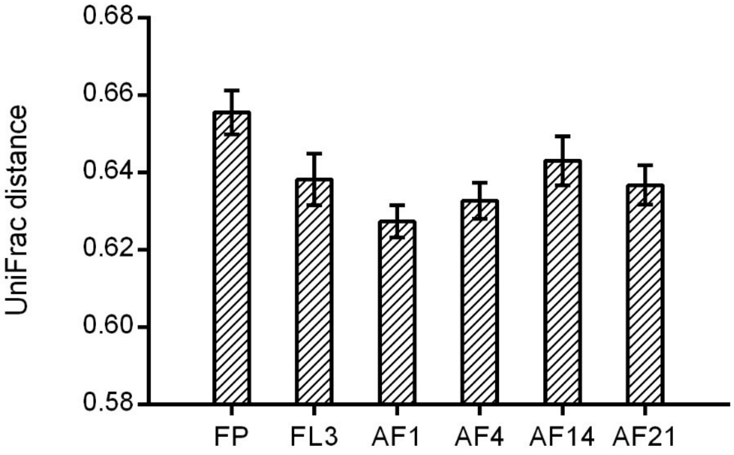

Supplementary Figure S2 UniFrac distance between bacterial communities on six sampling days. FP, representing spring fallow period; FL3, third day of flooding; AF1, 4, 14, 21 refer to the first, fourth, 14th, and 21st days after fertilization, respectively.

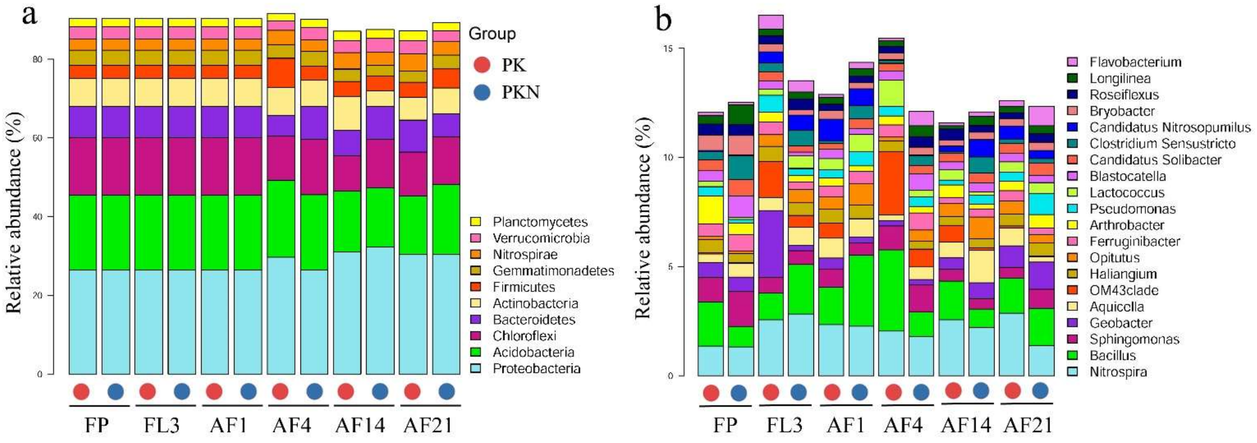

Supplementary Figure S3 Dominant bacterial populations and their composition: top 10 phyla (a) and top 20 genera (b) of all samples. FP, representing spring fallow period; FL3, third day of flooding; AF1, 4, 14, 21 refer to the first, fourth, 14th, and 21st days after fertilization, respectively.

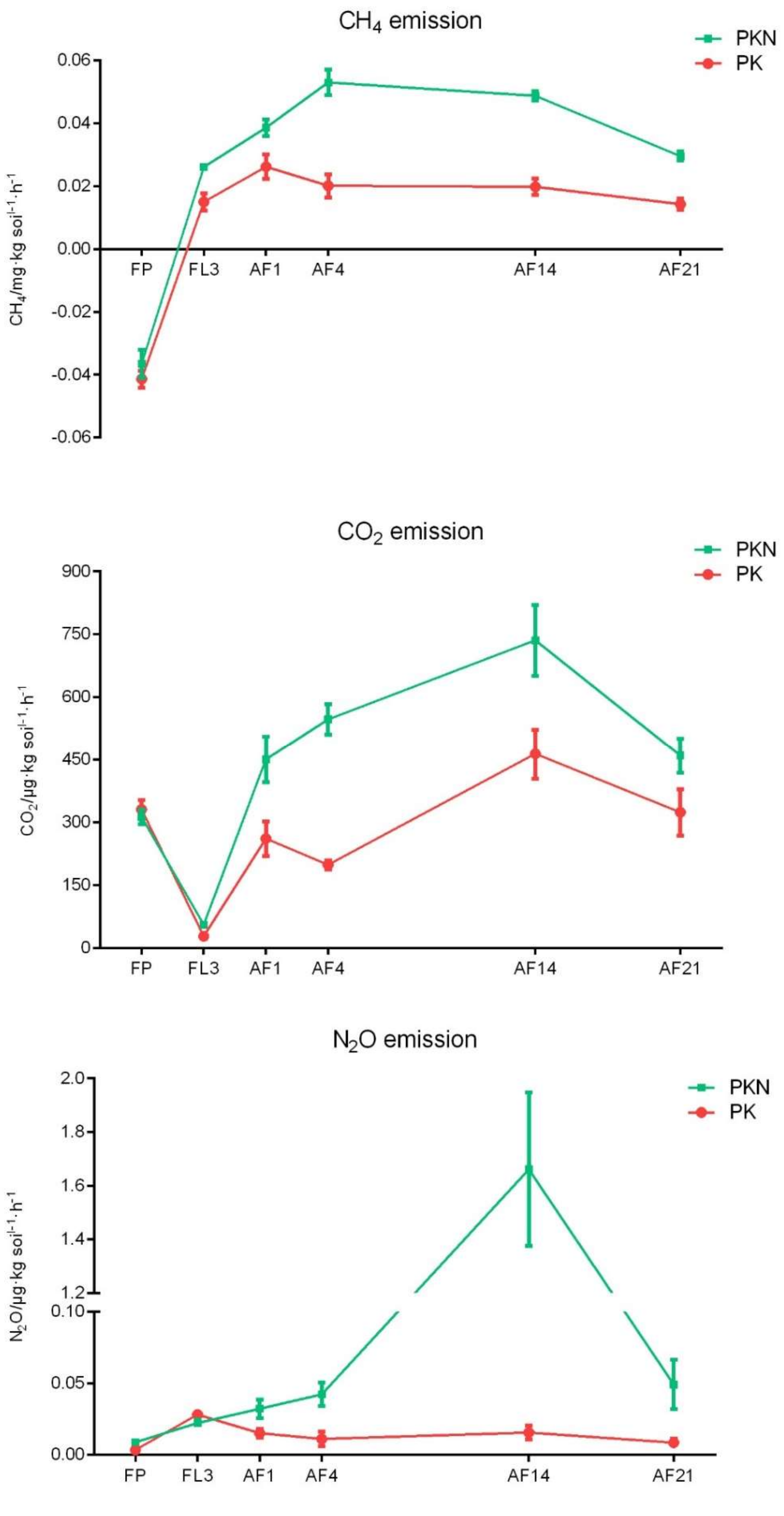

Supplementary Figure S4 Dynamics of CH_4, CO_2 and N_2O emissions of all samples. FP, representing spring fallow period; FL3, third day of flooding; AF1, 4, 14, 21 refer to the first, fourth, 14th, and 21st days after fertilization, respectively.

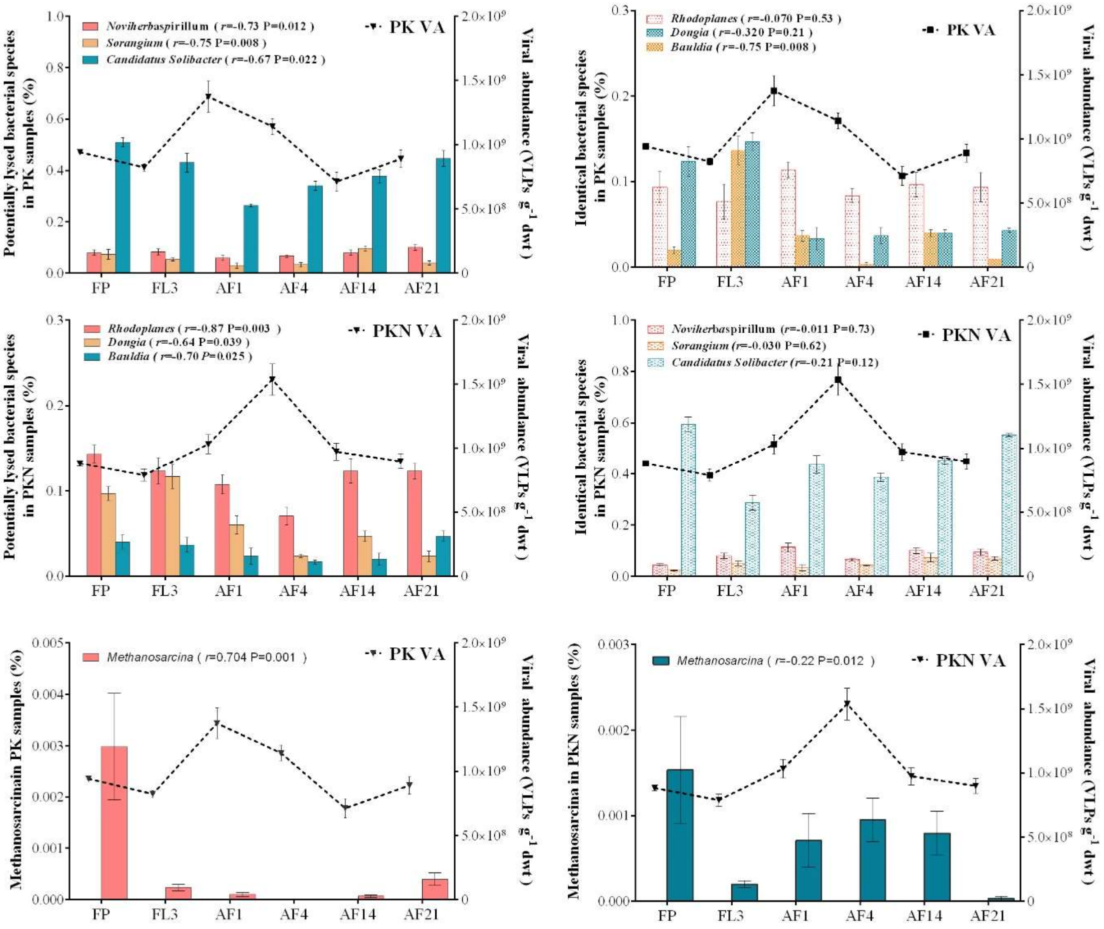

Supplementary Figure S5 In the CK group, dynamics of bacteria which are potentially lysed in the U group; and in the U group, dynamics of bacteria which in the CK group are potentially lysed. FP, representing spring fallow period; FL3, third day of flooding; AF1, 4, 14, 21 refer to the first, fourth, 14th, and 21st days after fertilization, respectively.
